# Blue Light Attracts More *Spodoptera frugiperda* Moths and Promotes Their Flight Speed

**DOI:** 10.3390/insects15020129

**Published:** 2024-02-14

**Authors:** Haibo Yang, Jing Lu, Pinhong Zhu, Yalan Sun, Zhenjie Hu, Dingxu Li, Jianrong Huang

**Affiliations:** 1 College of Horticulture and Plant Protection, Henan University of Science and Technology, Luoyang 471000, China; lujing981101@163.com (J.L.); phzhu@haust.edu.cn (P.Z.); yalansun@haust.edu.cn (Y.S.); zhenjiehu@163.com (Z.H.); dingxli@haust.edu.cn (D.L.); 2 Institute of Plant Protection, Henan Academy of Agricultural Sciences, Zhengzhou 450002, China

**Keywords:** *Spodoptera frugiperda*, sensitive wavelength, flight capability, light trap, light pollution

## Abstract

**Simple Summary:**

The fall armyworm, *Spodoptera frugiperda*, is an important migratory pest that causes severe crop damage. Light traps can monitor and control *S. frugiperda*. However, the black light traps that are currently used are less effective in trapping this pest. The efficiency of light traps is determined by the sensitive wavelength and the flight-to-light capacity. Our study found that *S. frugiperda* was more sensitive to blue light compared to other light. The flight speed of *S. frugiperda* varied significantly under different light conditions. In detail, with dark conditions as the control, the highest flight speeds and the largest percentage of fast-flying individuals were exhibited under blue light, whereas the lowest flight speeds and the smallest percentage of fast-flying individuals were displayed under ultraviolet (UV) light. Based on these results, we recommend the use of blue light traps for capturing *S. frugiperda* moths. These results provide theoretical support for improving the efficiency of light traps in controlling *S. frugiperda*.

**Abstract:**

Light traps are a useful method for monitoring and controlling the important migratory pest, the fall armyworm, *Spodoptera frugiperda*. Studies have shown that *S. frugiperda* is sensitive to blue, green, or ultraviolet (UV) light, but the conclusions are inconsistent. Furthermore, conventional black light traps are less effective for trapping *S. frugiperda*. To improve the trapping efficiency of this pest, it is crucial to determine the specific wavelength to which *S. frugiperda* is sensitive and measure its flight capability under that wavelength. This study investigated the effects of light wavelength on the phototaxis and flight performance of *S. frugiperda*. The results showed that blue light was the most sensitive wavelength among the three different LED lights and was unaffected by gender. The flight capability of *S. frugiperda* varied significantly in different light conditions, especially for flight speed. The fastest flight speed was observed in blue light, whereas the slowest was observed in UV light compared to dark conditions. During a 12 h flight period, speed declined more rapidly in blue light and more slowly in UV, whereas speed remained stable in dark conditions. Meanwhile, the proportion of fast-flying individuals was highest under blue light, which was significantly higher than under UV light. Therefore, the use of light traps equipped with blue LED lights can improve the trapping efficiency of *S. frugiperda*. These results also provide insights for further research on the effects of light pollution on migratory insects.

## 1. Introduction

The fall armyworm, *Spodoptera frugiperda* (Lepidoptera: Noctuidae), is an important migratory pest native to tropical and subtropical regions of the Americas [1]. The strong long-distance migratory ability of this pest has led to its rapid spread throughout the world. Since invading Africa in 2016, this pest has invaded 47 African and 18 Asian countries [2,3]. In 2020, it was also found in Australia and has now become a worldwide important agricultural pest [4]. As a polyphagous pest, *S. frugiperda* has a wide range of host plants, as many as 353 species from 76 families, including various food crops such as corn and rice [5]. *Spodoptera frugiperda* has been invading China since 2019 from Yunnan province and has rapidly spread to 27 provinces throughout the country, significantly threatening grain production in China [6]. Current control measures of *S. frugiperda* mainly rely on chemical pesticides. However, it has not only developed resistance to a variety of insecticides but also causes environmental pollution [7,8]. Therefore, there is an urgent need to find efficient and eco-friendly strategies to manage this pest.

Using insect phototaxis, light-trap technology is an important pesticide-free method in integrated pest management (IPM), especially in the monitoring and early warning of migratory pests [9]. Previous studies have shown that *S. frugiperda* can be monitored and trapped using light traps for population monitoring and control [10]. However, light trapping efficiency in the field was low [11,12], which was because the phototactic rate of *S. frugiperda* was significantly lower than that of other nocturnal moths such as *Helicoverpa armigera* [12,13]. Nevertheless, *S. frugiperda* flew to the light trap faster than *H. armigera*, which may be linked to the stronger flight ability of *S. frugiperda* under light [12]. Therefore, it is essential to investigate the flight capability of *S. frugiperda* in the presence of light to improve the trapping efficiency of light traps.

Phototaxis is an instinctive response formed by insects in the process of long-term evolutionary adaptation, and insects are sensitive to different wavelengths with different intensities. Since most insects contain ultraviolet (UV)-, blue- and green-sensitive photoreceptors, the sensitive wavelengths of insects are mainly concentrated in the UV, blue, and green light regions [14]. Different species of insects have different sensitive wavelengths [15]. Defining the sensitive wavelengths of pests and setting the target sensitive light source helps develop efficient, specific, and ecological security light trapping techniques [9]. As with most insects, existing studies indicated that *S. frugiperda* is sensitive to blue, green, or UV light, albeit with mixed results [12,13,16,17,18]. Insect spectral sensitivity can affect various life activities, including orientation, navigation, foraging, hunting, courtship, egg-laying, and other behavioral activities [19,20]. All of these life activities are related to the flight ability of insects. However, the flight capability of *S. frugiperda* under these sensitive wavelengths of light has not yet been studied.

In addition, with the rapid development of society and the increasing level of urbanization, especially the implementation of the urban “bright light project”, many cities are lit by artificial colorful lights at night, but these inevitably cause light pollution [21,22]. Light pollution has caused adverse effects on human health and the ecosystem and is also bound to affect the flight activities of nocturnal insects [23], especially migratory insects. Therefore, the influence of various wavelengths of color light pollution on *S. frugiperda* flight activities cannot be ignored.

Herein, we first compared the phototactic rate of *S. frugiperda* to blue, green, and UV light using a phototactic response device and determined the sensitive wavelength of *S. frugiperda*. We then investigated its flight capability under blue light, green light, UV light, and dark conditions using a tethered-flight mill system. Our results not only offer theoretical support for improving the trapping efficiency of *S. frugiperda* using light traps but also serve as a valuable reference for studying the effects of light pollution on the population dynamics of migratory insects.

## 2. Materials and Methods

### 2.1. Insects

The *S. frugiperda* eggs were procured from Henan Jiyuan Baiyun Industry Co., Ltd. (Jiyuan, China). A laboratory population was established after many generations of continuous rearing and breeding. Larvae were reared with an artificial diet [24] in a climatic chamber (HPG-280HX, Harbin Donglian Electronic Technology Development Co., Ltd., Harbin, China) at 25 ± 1 °C, 70% ± 5% relative humidity, and a 16:8 h (L:D) photoperiod. Pupae were sexed, males and females were kept separately, and emerged adults were fed with a 10% honey solution. Since 3-day-old *S. frugiperda* adults exhibited the strongest phototaxis and flight ability [17,25], we used 3-day-old moths to conduct the positive phototaxis and flight capacity experiments.

### 2.2. LED Light Sources

All LED lights (40 W) were purchased from Xuzhou Ai Jia Electronic Technology Co., Ltd. (Xuzhou, China). The wavelength of UV (365–370 nm), blue (465–470 nm), and green (520–530 nm) lights were selected according to previous reports, and the light intensities were measured by a digital lux meter (Smart Sensor, AS813, Dongguan Wanchuang Electronic Products Co., Ltd., Dongguan, China).

### 2.3. Phototactic Behavior Chamber

**Figure 1:**
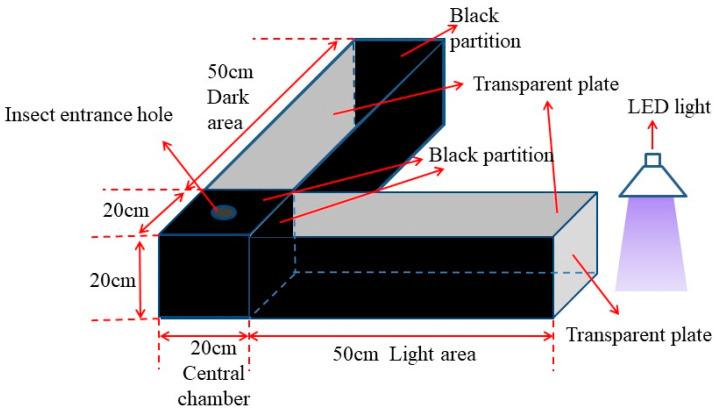
Schematic diagram of phototactic response test chamber.

The phototactic behavior chamber was modified according to the experimental devices by Kim et al. (2018) [26]. In brief, the device consists of three parts: the light chamber, the dark chamber (20 cm × 20 cm × 50 cm), and the cube box (20 cm × 20 cm × 20 cm), and these were combined at right angles to each other (Figure 1). Two transparent plates above the light and dark chambers were utilized for observing the moths’ behavior. An insect entrance hole was opened at the center of the cube box, and a transparent plate was installed on the outside of the light area for transmitting light, as well as blocking heat and odors. The light source was placed at a suitable height above the outside of the light chamber, and the light intensity was adjusted using a resistor.

### 2.4. Phototactic Behavior Test

The phototactic responses of the moths were tested in a chamber within a dark room. Before the experiment, test moths were released into the cube box through the entrance hole and were allowed to adapt to the darkness for 30 min. Each test started at 8:00 p.m. [12,13]. The upper part of the two behavior chambers was wrapped in opaque black cloth during the experiments. The light intensity at the end of the light chamber was 100 lux, and the number of adults in the light chamber was counted 30 min after the light was turned on. Phototactic rate (%) = (number of moths in the light chamber/total number of moths) × 100. Both 3-day-old female and male moths were checked individually for examining the phototactic responses to the LED lights with different wavelengths. Each treatment was repeated for at least three repetitions with 15 moths each time.

### 2.5. Flight Capability Determination

The flight performance of *S. frugiperda* moths was measured by an 8-channel flight mill system, following the method used by Chen et al. (2022) [27]. Our experiments were carried out in a completely dark flight chamber covered with blackout materials. Only healthy, undamaged moths were tested in the flight mill. For each replicate, a moth was sedated at 4 °C for a minimum of 2 min before tethering. Scales were gently removed from the protergum with a fine brush. Individual moths were attached to the end of the flight mill arm using a small droplet of adhesive glue (Deli Group Co., Ltd., Ningbo, China). The LED light sources were suspended centrally above the flight mills, and the light intensity was 100 lux at the position where the moth was hung on the flight arm. Tethering was completed 30 min before the test. Each test was conducted for 12 h (from 20:00 to 08:00), and all tests were conducted under optimum conditions at 25 ± 1 °C, and relative humidity was 70% ± 5%. The recordings of flight parameters (flight distance, total flight duration, flight speed, and maximum flight speed) were performed automatically by a computer. A moth was considered a replicate, and at least 20 moths were tested for each treatment.

Both 3-day-old female and male moths were used for the experiments examining the flight capacity under different wavelengths of LED light. Dark conditions were used as a control. For revealing the effect of mating status, because *S. frugiperda* adults in blue light exhibited strong flight performance, 64 virgin unmated adults and 60 mated adults (3-day-old) in blue light were used.

### 2.6. Statistical Analysis

Before the analysis, all data were tested for normal distribution (Shapiro–Wilk test) and variance homogeneity (Levene’s test). The phototaxis data, flight distance, and flight speed data were normally distributed; Student’s *t*-test was used for the comparison of pairs; and ANOVA was used for different groups followed by Tukey’s honestly significant difference (HSD) post hoc test (*p* < 0.05). Flight duration data were not normally distributed, despite various transformation trials. The Mann–Whitney U test was used for pairwise comparisons, and the Kruskal–Wallis test was used for multiple comparisons. A Chi-square test was further used to compare the proportion of flight speed types under different light conditions. Statistical analyses were performed with IBM SPSS Statistics 25 software (SPSS Inc., Chicago, IL, USA).

## 3. Results

### 3.1. Phototactic Rates of S. frugiperda to Different Light Sources

**Figure 2:**
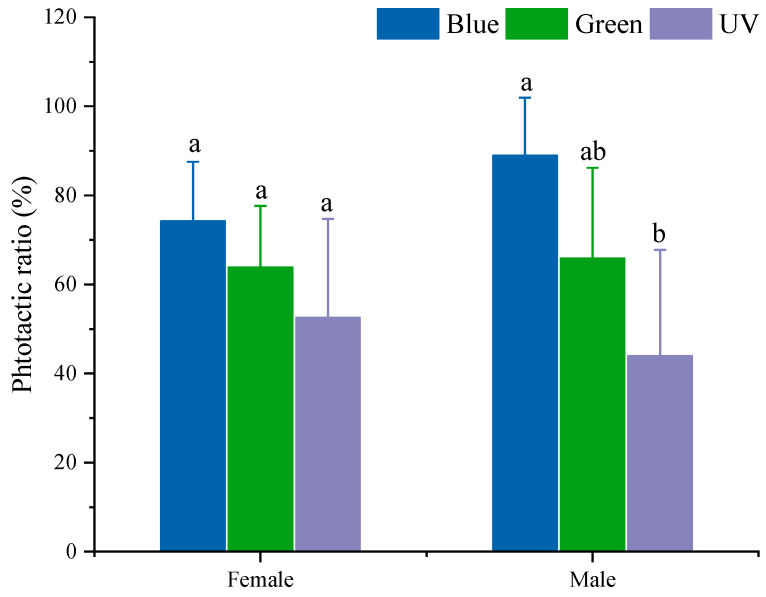
Phototactic rates of *S. frugiperda* to different wavelength lights. Data are mean ± SE. Different letters above the bars within the same gender indicate significant differences according to Tukey’s HSD test (*p* < 0.05). Blue, blue LED; Green, green LED; UV, UV LED.

On the whole, the phototactic rates of *S. frugiperda* to the three wavelengths of light decrease in the following order: blue light > green light > UV light (Figure 2). Statistically, for females of *S. frugiperda*, there were no significant differences between the three light sources (F_2,20_ = 2.723, *p* = 0.09). Additionally, male moths exhibited a significantly stronger attraction to blue light than UV light (F_2,12_ = 5.665, *p* = 0.023), with no significant difference between blue light and green light.

In addition, there were no obvious differences in the phototactic rates between the sexes of *S. frugiperda* to the same light source, though the phototactic rates of males to blue light and green light were slightly higher than those of females (Figure 2).

### 3.2. Flight Performance of S. frugiperda in Different Light Conditions

**Figure 3:**
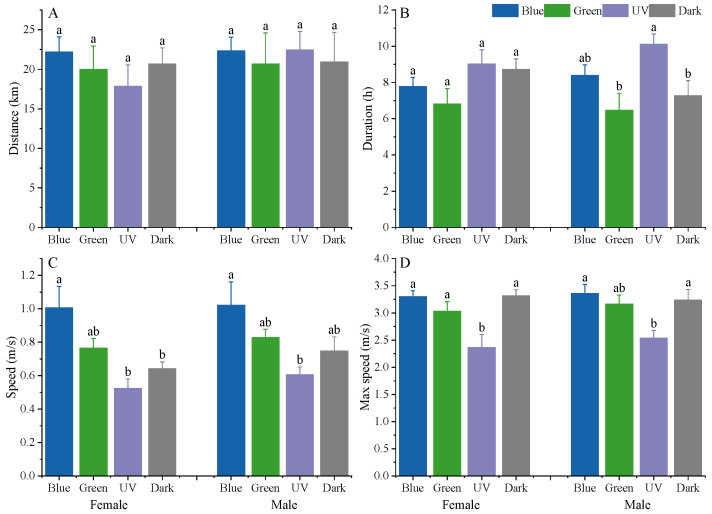
Flight distance (**A**), flight duration (**B**), flight speed (**C**), and maximum flight speed (**D**) of *S. frugiperda* in different light conditions. Different letters above the bars within the same gender indicate significant differences between different treatments (*p* < 0.05), based on the results of Tukey’s HSD test for flight distance, speed, and maximum speed or Kruskal–Wallis test for flight duration. Blue, blue LED; Green, green LED; UV, UV LED; Dark, dark conditions.

There was no significant difference in the flight distance of both female and male moths in different light conditions (females: F_3,77_ = 1.456, *p* = 0.235, males: F_3,90_ = 0.111, *p* = 0.954; Figure 3A). Both female and male moths had the longest flight duration under UV light, although females did not exhibit significant differences among treatments (χ^2^ = 6.093, df = 3, *p* = 0.107, Figure 3B). Male moths in UV light showed a significantly longer flight duration than those in green and dark conditions (χ^2^ = 11.061, df = 3, *p* = 0.011; Figure 3B), while it was not different from those under blue light.

Both female and male moths fly fastest in blue light and slowest in UV light (Figure 3C). The flight velocity of female moths in blue light was significantly faster than those in UV and dark conditions (F_3,108_ = 4.942, *p* = 0.003); the flight speed of male moths in blue light was faster than that in UV light (F_3,90_ = 3.246, *p* = 0.027), whereas no significant difference was found with green light and dark conditions.

Similar to flight speed, the maximum flight speed of both female and male moths was lowest under UV light (Figure 3D). The maximum flight speed of females under UV light was significantly lower than that of the other three light conditions (F_3,108_ = 8.163, *p* < 0.001), and males had significantly lower maximum flight speeds under UV light compared to blue light and dark conditions (F_3,90_ = 5.574, *p* = 0.002) but were not significantly different from that of the green light.

**Figure 4:**
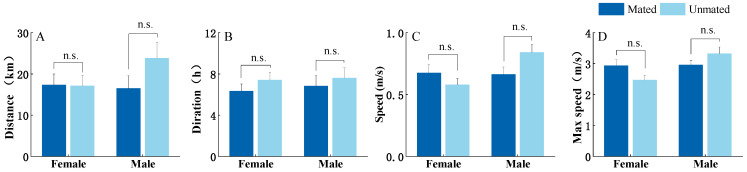
Comparison of flight performance between mated and unmated *S. frugiperda* in blue light. (**A**) flight distance, (**B**) flight duration, (**C**) flight speed, and (**D**) maximum flight speed. Means between the two groups within the same gender were compared according to Student’s *t*-test (*p* < 0.05). “n.s.” = not significant.

In addition, there was no significant difference in flight parameters between females and males. The mating status also did not affect the flight parameters of *S. frugiperda* females and males (*p* > 0.05, Figure 4). Therefore, the flight speeds of both sexes were combined and not compared separately later.

### 3.3. Flight Speed Dynamics in Different Light Conditions

**Figure 5:**
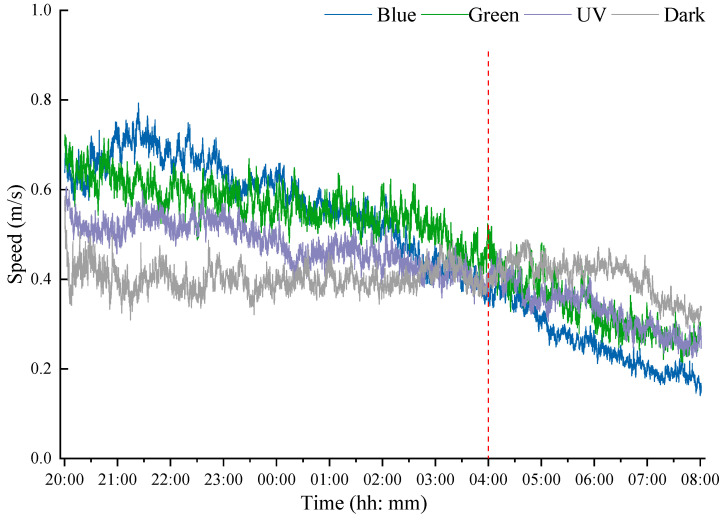
Flight speed changes of *S. frugiperda* under different light conditions. The values presented are the average speeds of all test moths in each group at 5 s intervals. The red dotted line indicates 4 a.m. Blue, blue LED; Green, green LED; UV, UV LED; Dark, dark conditions.

Over a 12 h assessment period, the flight velocity of *S. frugiperda* under different wavelengths of light gradually decreased but remained stable in dark conditions (Figure 5). The largest decline in flight speed was observed in blue light, ranging from 0.79 m/s to 0.14 m/s (slope of −1.18). The speed also declined in green light (slope of −0.80) from 0.72 m/s to 0.21 m/s, and in UV from 0.61 m/s to 0.22 m/s (slope of −0.56). However, it remained consistent around 0.40 m/s in the dark. Interestingly, the change curve of flight speed under different treatments had a clear crossover at about 04:00 a.m. During the first 8 h flights (08:00 p.m.–04:00 a.m.), flight speeds followed the order of Blue > Green > UV > Dark in all four treatments; however, the order was reserved during the remaining 4 h (04:00 a.m.–08:00 a.m.).

### 3.4. Flight Speed Types in Different Light Conditions

**Figure 6:**
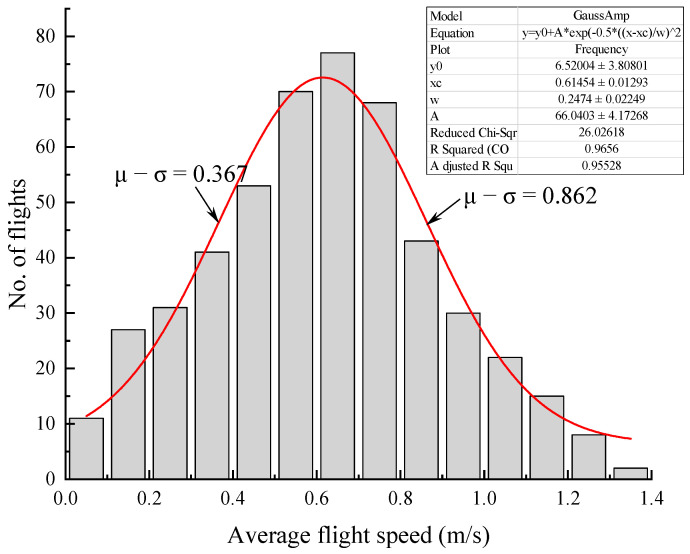
Frequency distribution of total flight speed of *S. frugiperda* moths in the 12 h of testing. The equation and Gaussian fitting curve are indicated on graph; the left and right arrows indicate the location of μ − σ and μ + σ, respectively. μ and σ are the mean and standard deviation values for the normal distribution.

To further compare the flight speeds under different light conditions, we analyzed the frequency distribution of flight speeds for all moths subjected to testing. Flight speed was normally distributed with mean μ = 0.615 (mean speed, df = 199) and σ = 0.247 (Figure 6), which means 68.26% of the flight speeds lie within ± 0.247 m/s. Accordingly, 0.35 m/s (μ − σ = 0.367 m/s) and 0.85 m/s (μ + σ = 0.862 m/s) can be used as cut-off values for slow-, medium-, and fast-speed individuals.

**Figure 7:**
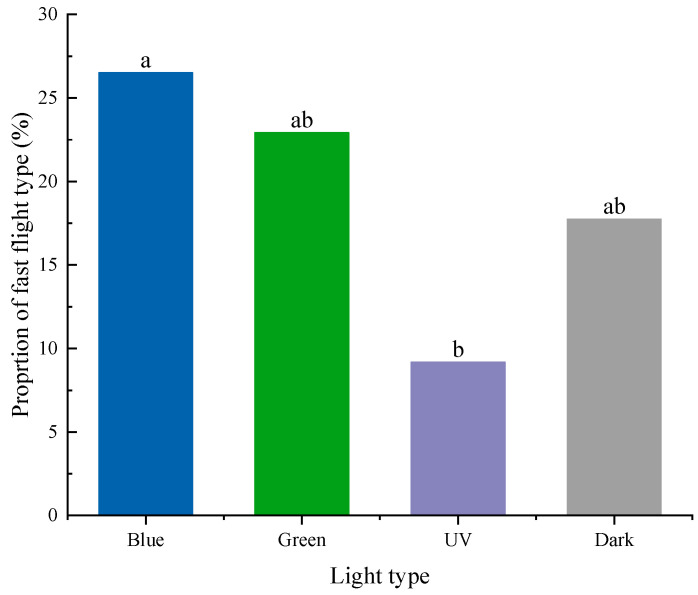
Proportions of individuals with faster flight speed under different light conditions. Different letters above the bars indicate significant differences between different treatments (*p* < 0.05), based on the results of the chi-square test.

Therefore, based on the criteria above, the flight speeds in the four light conditions were divided into slow, medium, and fast types. The distribution proportion of individuals differentiated by flight speed varied significantly among the four light conditions (χ^2^ = 12.569, *p* = 0.006). In detail, the proportion of fast-flying individuals was highest under blue light (26.51%), which was significantly higher than the proportion of those under UV light (9.18%) and not significantly different from those under green light (22.92%) and in dark conditions (17.74%) (Figure 7).

## 4. Discussion

Vision plays an important role in insect behavior. As the basis of visual search, light can affect various life activities of insects. Different wavelengths of light produce varying responses in insects [15]. Understanding the flight capacity of pests in varying light spectra can assist in enhancing the trapping efficiency of light traps. In this study, *S. frugiperda* showed the highest phototactic rates to blue light when compared to green and UV light. Moreover, different wavelengths of light also can impact the flight performance of *S. frugiperda*, particularly its flight velocity. When compared to dark conditions, *S. frugiperda* exhibited the fastest flight under blue light and the lowest flight under UV light. Sex and mating status had no significant effect on the flight performance of *S. frugiperda*. Flight speeds under blue, green, and UV light decreased gradually over time, whereas flight speed in dark conditions remained constant. In addition, the proportion of fast-flying individuals was the highest under blue light, whereas it was the lowest under UV light. Considering the results above together, we recommend using light traps equipped with blue LED light to improve the efficiency of trapping *S. frugiperda* moths.

Many nocturnal insects exhibit positive phototaxis to artificial light, light traps are widely used to monitor and manage pest populations [9,28]. Many insects, including moths, have three photoreceptors that sense light in the blue, green, and UV wavelength ranges [14,29,30]. The sensitivity of insects to light varies by species [14,15]; even for the same insect, wavelength sensitivity varies depending on some biological or abiotic factors [31,32]. The results of this study showed that *S. frugiperda* was more sensitive to blue lights, which was consistent with the report of Wang et al. (2022) [13]. Some other insects have also been reported to be sensitive to blue light and can be captured by the blue LED traps, such as the white-backed planthopper (*Sogatella furcifera*), brown planthopper (*Nilaparvata lugens*), rice weevil (*Sitophilus oryzae*), angoumois grain moth (*Sitotroga cerealella*), and thrips [28,33,34]. However, our results were inconsistent with the results of other studies; Zhang et al. (2023) found that *S. frugiperda* preferred UV light [18], and Nascimento et al. (2018) and Liu et al. (2023) reported that *S. frugiperda* preferred green light [16,17]. Potential reasons for this discrepancy could include variations in the type of light source, light intensity, duration of light treatment, and the physiological status of the insect [31,32]. More importantly, the use of a single wavelength or multiple wavelengths in determining sensitive wavelengths can significantly affect the results [32].

Since UV light or light sources with high UV content are usually more attractive to insects [35], it has been taken for granted that traditional black light should be used to control and monitor various pests. However, field experiments have shown that black lights were less effective in trapping *S. frugiperda* moths; only a few or even individual moths were captured in the wild [11,12,36]. The phototactic behavior of *S. frugiperda* to UV light was weaker than that of *H. armgiera*, which was further confirmed by indoor light trap capture experiments and opsin expression levels [12,13]. However, *S. frugiperda* moths can fly faster to the light than *H. armgiera* moths [12]. Our results also revealed that *S. frugiperda* moths exhibited faster flight speeds in light conditions, especially during the first 8 h flight. Insects exhibit faster flight speeds in the light region for two reasons: positive phototaxis, which attracts them to the light source, and negative phototaxis, which causes them to escape from the light source. This study is apparently due to positive phototaxis promoting the flight speeds of *S. frugiperda*.

Within 12 h of the flight, the flight speed of *S. frugiperda* gradually decreased in bright light conditions, while its flight speed remained stable in the dark, indicating that light did have an impact on flight speed. Furthermore, during the first 8 h of flight, the flight speed in the light environment consistently exceeded that of flight speed in the dark. However, by the subsequent 4 h of flight, the speed in the light was already significantly lower than that in the dark. This is due to the moth’s phototaxis, which is highly active during the early stages of flight but difficult to maintain for a long time. As the flight progresses, the speed gradually decreases, and a faster initial speed results in a faster rate of decline. Considering the limited trapping range of the light traps in the field [13], moths can reach the traps with a short rapid flight. Therefore, the blue LED light source is commended to improve trapping efficiency.

As urbanization progresses and the use of artificial light also increases, the darkness of night-time to which most animals are adapted is threatened by increasing global light pollution [37,38]. Light pollution not only affects potential human health but also severely impacts birds, aquatic animals, and terrestrial animals, especially nocturnal insects [39,40,41]. Light pollution can have deleterious effects by disrupting the journeys of migratory insects, as many nocturnal species rely on compass information in the sky to hold their course [42,43]. For *S. frugiperda* with a strong long-distance migration ability, nighttime migration and dispersal will inevitably be affected as well. This study indicates that the flight performance of *S. frugiperda* was affected in light environments that are sensitive to its phototactic response, especially the flight speed. The ubiquitous presence of artificial light may alter their migratory paths, resulting in localized outbreaks, or may increase their dispersal, resulting in a large outbreak occurring. More importantly, the sky glow, which extends far beyond urban areas, can, in some instances, have a more profound effect than the direct illumination of light pollution [44]. The sky glow can influence population dynamics through effects on the behavior of individuals, such as general activity and circadian cycles, which, in turn, can influence the community composition and ecosystem structure and processes [41]. In addition, we only studied the phototaxis and flight behaviors of *S. frugiperda* in monochromatic light sources. However, it should be noted that in general, light pollution in urban areas is mixed with multiple light sources. Therefore, whether different effective light sources have synergistic effects to produce more trapping efficiency is to be further investigated.

## 5. Conclusions

*Spodoptera frugiperda* had the highest phototactic rate for blue light and had the fastest flight speeds under blue light but the slowest speeds under UV light, compared to the darkness. Sex and mating status did not affect their flight performance. In conclusion, *S. frugiperda* flight activity was the most active in the most sensitive blue light. The results of this study suggest that the use of trapping lamps with blue LED light sources may improve the efficiency of trapping *S. frugiperda* and also provide insights for further research on the effects of increasing light pollution on migratory insects.

## Data Availability

The data presented in this study are available in the article.
